# Associations Between Male Partner Participation, Women’s Autonomy, and Postpartum Care Seeking Among First Time Mothers, 15–24 Years, in Kinshasa, Democratic Republic of the Congo: A Cross-Sectional Analysis

**DOI:** 10.3390/ijerph23081061

**Published:** 2026-08-15

**Authors:** Madeline Woo, Anastasia J. Gage, Julie H. Hernandez, Pierre Z. Akilimali, Jane T. Bertrand

**Affiliations:** 1 Department of Health Policy and Management, Celia Scott Weatherhead School of Public Health and Tropical Medicine, Tulane University, 1440 Canal St., New Orleans, LA 70112, USA; 2 Department of International Health and Sustainable Development, Celia Scott Weatherhead School of Public Health and Tropical Medicine, Tulane University, 1440 Canal St., New Orleans, LA 70112, USA; agage@tulane.edu (A.J.G.); jhernan7@tulane.edu (J.H.H.); bertrand@tulane.edu (J.T.B.); 3 Department of Biostatistics and Epidemiology, Kinshasa School of Public Health, University of Kinshasa, Kinshasa P.O. Box 11850, Democratic Republic of the Congo; pierretulanefp@gmail.com

**Keywords:** postpartum care, postnatal care, male partner involvement, first-time mothers, adolescent mothers, young mothers

## Abstract

**Highlights:**

**Public health relevance—How does this work relate to a public health issue?**

- The postpartum period is an important time to identify danger signs; however, postpartum visit attendance in the Democratic Republic of the Congo (DRC) is low at only 30%.
- Young mothers and their neonates are at increased risk of adverse outcomes; therefore, identifying strategies to address this population is a priority for public health.

**Public health significance—Why is this work of significance to public health?**

- This study tests two programmatic strategies to increase postpartum care utilization among first-time mothers, 15–24 years, in Kinshasa, DRC.
- The findings suggest that the type of male participation matters; male partner involvement during pregnancy and delivery was associated with negative outcomes in postpartum care utilization while male partner involvement in childcare was positively associated with the same outcome.

**Public health implications—What are the key implications or messages for practitioners, policy makers and/or researchers in public health?**

- Policy makers should carefully contextualize programs aimed at increasing male partner involvement among young first-time mothers to avoid adverse unintended consequences.
- Formative research on what types of male partner involvement are most effective and what autonomy means to adolescent and young mothers is needed.

**Abstract:**

The Democratic Republic of the Congo (DRC) has high maternal and neonatal mortality rates and only a third of mothers and newborns have a postpartum or postnatal care visit. Increasing male participation and enhancing women’s autonomy are strategies to increase maternal healthcare utilization, but their applicability to first-time mothers (FTMs) in Kinshasa, DRC is unknown. We conducted a secondary analysis using cross-sectional survey data from a maternal and child health project among FTMs and their male partners in Kinshasa. The sample consisted of 1750 FTMs aged 15–24 years. The outcome variables were (1) a postpartum care visit within six weeks of facility discharge for the mother, (2) a postnatal care visit within six weeks of facility discharge for the neonate, and (3) either a postnatal or postpartum care visit within six weeks of facility discharge. Two logistic multivariate regression models were run for each outcome. The first model included all explanatory and demographic variables, and the second model added two interaction terms to test for mediation by in-union status. The male participation in maternal care index was negatively associated with all outcomes and the male participation in childcare index was positively associated with all outcomes. In-union status moderated the relationship between the male involvement indices and postpartum care for the mother but not for postnatal care for the newborn. Our findings show that the type of male participation may be meaningful for increasing postpartum and postnatal care-seeking and male partner participation initiatives need to be contextualized to prevent adverse outcomes.

## 1. Introduction

The postpartum period, defined as the first 42 days following birth [1], is a key time in the health of both mother and newborn. Over half of maternal deaths occur after childbirth [2] and neonates (<28 days) make up the largest share of under-five deaths worldwide [3]. The World Health Organization (WHO) recommends at least four postpartum or postnatal visits for all mothers and newborns within six weeks of delivery [1]. Low- and middle-income countries (LMICs) have disproportionally high rates of maternal and neonatal mortality, with sub-Saharan African countries reporting the highest rates [4]. The first six weeks after delivery represent a crucial moment for intervention to reduce these rates.

Despite the importance of postpartum care (PPC) for mothers and postnatal care (PNC) for newborns, the postpartum period is an understudied and neglected component of maternal health coverage [1,5,6]. Low PPC attendance is a global issue, with 40% of women in the United States not attending a postpartum visit [5], and attendance rates are especially low among low-income women [7]. PPC utilization among women in sub-Saharan Africa varies, ranging from 86% in Cameroon to 8% in Ethiopia [8], and a systematic review found a paucity of literature on care seeking for neonates outside of the South Asian context [9].

Maternal care utilization in the Democratic Republic of the Congo (DRC) reflects global programmatic trends in the maternal healthcare continuum, with high antenatal care (ANC) attendance and facility delivery and low PPC and PNC utilization [10]. Nationally, about 85% of women 15–44 years had at least one ANC visit during their last pregnancy, and of those who had an ANC visit, 46% had four or more visits [11]. Facility delivery is 83% nationally, and 97% in the capitol of Kinshasa [11]. Despite high ANC utilization and facility delivery, the DRC has the 22nd highest [12] maternal mortality ratio (693 deaths per 100,000 live births) [13] and the 24th highest [14] neonatal mortality rate (27 deaths per 1000 live births) worldwide [14]. Low PPC and PNC attendance may contribute to high maternal and neonatal mortality nation-wide as less than a third of women (29%) reported receiving PPC within two days of birth, though Kinshasa reported a higher rate (61%) [11]. PNC utilization was similar with only 32% of women in the DRC reporting that their neonate had a postnatal check-up within two days of birth [11].

Increased postpartum and postnatal care seeking could especially benefit adolescent mothers (10–19 years) as they have a higher risk of eclampsia, puerperal endometritis, and systemic infection and their newborns are at increased risk of preterm birth and low birth weight [15]. A study of maternal mortality in Eastern DRC found that mothers 19 years and younger had an increased risk of maternal mortality compared to mothers 20–39 years [16]. This is especially problematic as adolescents and young women in the DRC have high fertility rates, at 101.0 births per 1000 women for women 15–19 years and 202.9 births per 1000 women for women 20–24 years [17]. Nationally in the DRC, the share of adolescents (<20 years) and their neonates who received care within 42 days of birth was is similar across age cohorts at roughly two-thirds [11].

Male partner involvement is one initiative used to improve health outcomes for both mother and newborn in the postpartum period [2,18,19,20]. A systematic review of male partner involvement and maternal health outcomes in LMICs found that male involvement in both the antenatal and postnatal periods improved maternal health; however, attendance during delivery was not associated with improved health outcomes [19]. Inclusion of men in antenatal health education sessions in Nepal was associated with improved maternal outcomes and postpartum care seeking among women [21], while deference to husbands, in-laws, and community members was associated with delays in care-seeking [22]. The majority of studies on male participation and maternal health outcomes took place in South and East Asia [18,19]. In the DRC, male involvement can adversely impact women’s reproductive healthcare utilization; studies found that women’s agency and access to resources for family planning were impeded by their husbands’ role as the primary decision-maker [23,24].

Overall, there is a paucity of research on the relationship between male involvement and newborn health in LMICs and findings vary between contexts [20,25]. One study in Turkey found that male involvement increased early initiation of breastfeeding and improved the continuation of exclusive breastfeeding [26], while in India male involvement was associated with early initiation of breastfeeding but a discontinuation of exclusive breastfeeding at six months [27]. In South Africa there was an observed decrease in stillbirths and early neonatal mortality following a facility-based education initiative for male partners [28], while no effect was found in India [27] or Pakistan [29].

There has been limited research on male involvement and postpartum health outcomes in sub-Saharan Africa [19,30]. One study in Northwest Ethiopia found that 20% of fathers interviewed were involved in postnatal service based on a ten-point scale of male participation, but the study did not measure the association with any maternal or child health outcomes [31].

Increasing women’s autonomy [32,33], defined as the ability to obtain information and make decisions free from the control of others [34,35,36,37] is another strategy for increasing PPC and PNC utilization in sub-Saharan Africa [32]. Gender inequality in the DRC is high, with the DRC ranked 152nd out of 166 countries [38] on the Gender Inequality Index. Although the relationship between women’s autonomy and PPC and PNC utilization is not established in the DRC, previous findings on the association between women’s autonomy and reproductive and child health outcomes have been mixed [39,40,41,42,43,44]. Differences in study outcomes may be attributed to differences in how autonomy was measured. Ideally, male partner involvement and increasing women’s autonomy work synergistically to improve maternal and child health outcomes. However, care must be taken so that existing gender balances are not exploited and men’s involvement does not negate women’s preferences [45].

Both male partner involvement and women’s autonomy are multidimensional constructs that cannot be fully captured by a single measure. Women can simultaneously have high autonomy in one dimension and low in another [46,47], and male involvement has been operationalized in multiple ways [19]. Different dimensions may influence health behaviors through different mechanisms and therefore examining them separately may provide a better understanding of their relationships to PPC and PNC utilization.

This paper primarily examines the relationship between two dimensions of male partner engagement, involvement during pregnancy and delivery and participation in childcare, and timely PPC and PNC utilization among first-time mothers (FTMs) aged 15–24 years in Kinshasa. As male partner involvement can be impacted by marital status [48], we test for moderation by relationship status. This paper also explores the association between women’s autonomy and timely PPC and PNC utilization among FTMs aged 15–24 years in Kinshasa, DRC. Finally, we seek to examine if factors associated with timely care seeking after childbirth differ by recipient (mother or child).

## 2. Materials and Methods

### 2.1. Data

Secondary data were drawn primarily from the 2020 Momentum project endline survey, with demographic characteristics and some explanatory variables from the 2018 baseline survey. Momentum was a gender-transformative integrated family planning, maternal and newborn health, and nutrition project in Kinshasa. Pairs of nursing students delivered the intervention to FTMs aged 15–24 years and their male partners through home visits, community dialogue, and support group education sessions. The project was implemented in three health zones (Kingasani, Lemba, and Matete), and three comparison health zones (Bumbu, Ndjili, and Masina1) were selected based on similar demographic characteristics (e.g., ethnicity, socioeconomic status, education, population density, and access to services). Nulliparous FTMs were recruited at six-months gestation in all health zones and were identified by healthcare providers at facilities or recruited through community outreach.

Sample size was calculated based on detecting a 10-percentage point difference in timely initiation of postnatal care with 99% confidence and 99% power, an assumed attrition rate of 25%, and a design effect of 2.0 assuming a different sample design other than a cohort study. The estimated prevalence of newborns’ first prenatal check was 6.5% based on the 2013–2014 DRC Demographic and Health Survey (DHS). A sample size of 1213 FTMs was estimated for intervention health zones and the same amount for comparison health zones. Both the baseline and endline surveys were administered in all health zones.

Attrition between the baseline and the endline surveys was 20.7% and was similar in the comparison and intervention health zones. A total of 1927 FTMs completed the endline survey out of 2316 eligible FTMs. The Momentum study was conducted in accordance with the Declaration of Helsinki and approved by the Tulane University Institutional Review Board (1112188, 11 July 2018) and the University of Kinshasa School of Public Health Ethics Committee (ESP/CE/066/2018, 15 June 2018; ESP/CE/08/2020). We have received permission to use the data from the project PI and research manager.

### 2.2. Sample

The analytical sample comprised all FTMs aged 15–24 years who responded to the questions “Did anyone check on your health after you left the facility?” and “Did any health care provider, community health worker, or traditional birth attendant check on [CHILD’S NAME]’s health in the two months after you left [the facility]?” FTMs who did not have a facility delivery, were missing data for either outcome variable, or were missing data on important sociodemographic characteristics were excluded from analysis. Women who had a stillbirth were excluded from the sample because they were not asked about the timing of a PPC after facility discharge. A total of 1750 FTMs were included in this study.

### 2.3. Variables

Postpartum care within six weeks of facility discharge: This variable was based on FTMs’ responses to the questions “Did anyone check on your health after you left the facility?” and “How long after delivery did that check take place?” The timing of the postpartum checks after facility discharge was recorded as hours, days, or weeks. Timely PPC visits were coded as “1” if the FTM reported having a PPC visit within six weeks of facility discharge. The six-week period was defined based on the reported time period between discharge and the PPC visit. Six weeks was used as the cutoff based on current WHO recommendations for postpartum and postnatal care [1]. This definition does not include postpartum checks while at the facility for delivery as 93.0% of the sample reported having a postpartum check before hospital discharge.

**Figure A1:**
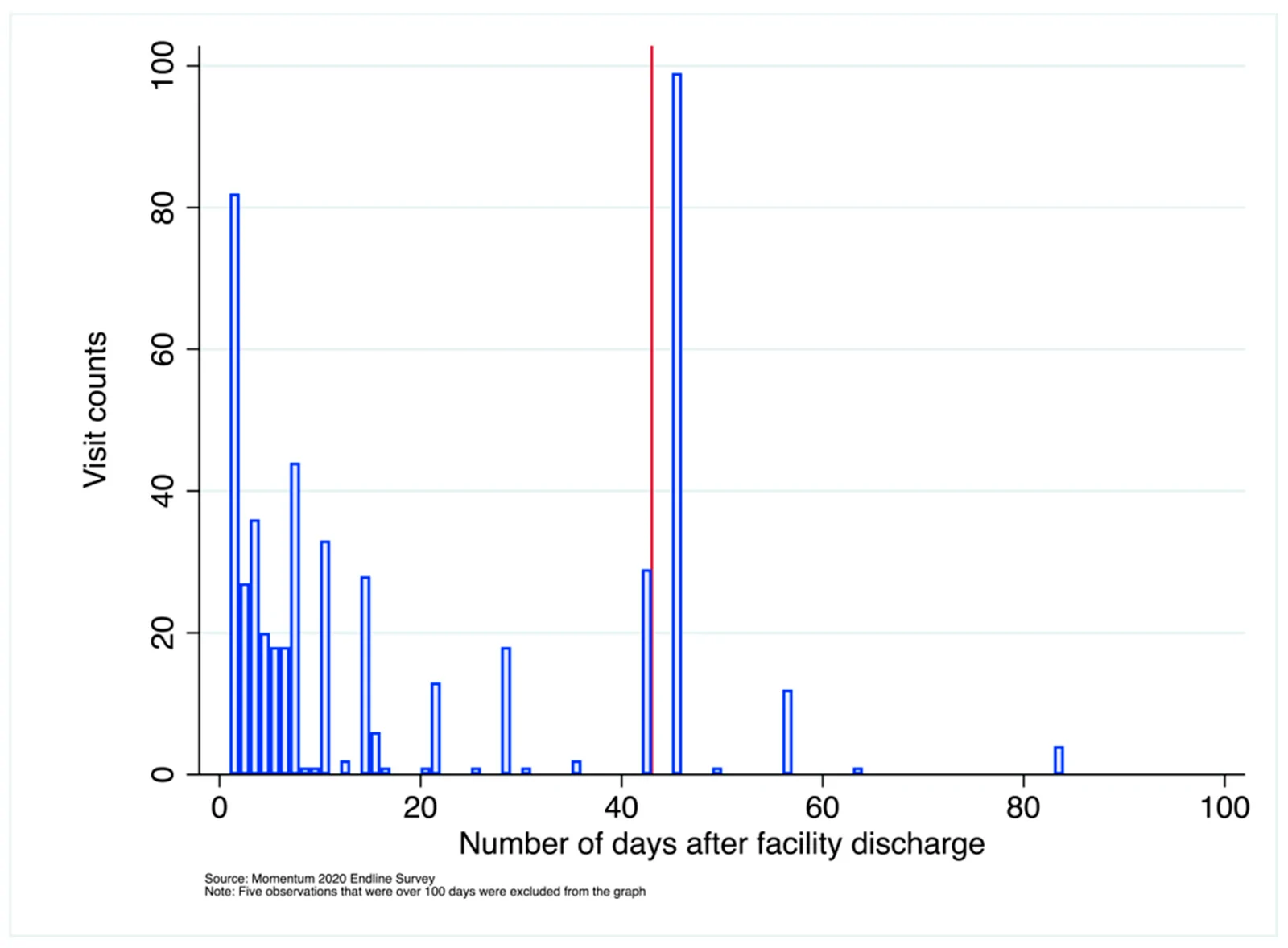
Number of postpartum care visits per day after facility discharge among FTMs aged 15–24 years old, Kinshasa, Democratic Republic of Congo. Red line denotes 42 days. The figure was created by the authors.

Analysis of PPC visit timing showed that there was possible heaping at 45 days, with 18.9% of FTMs who received a PPC visit reporting that a health care provider, community health worker, or traditional birth attendant checked on their health on the 45th day after facility discharge (Appendix B Figure A1). The endline survey was administered 18 months after delivery so recall bias may have led to “rounding”, meaning respondents were more likely to answer with numbers ending in “5” or “0” [49]. Additionally, forty-five days corresponds with a month and a half, and since time was not recorded in months, it is plausible that a woman who had a timely PPC reported it as “a month and a half” and her response was recorded as 45 days. Based on these factors, reporting error was viewed as a plausible explanation for the heaping at 45 days. Thus, the postpartum outcome variable was coded to include women who reported having a PPC at 45 days as receiving a timely PPC. All respondents who reported that they did not have a PPC after facility discharge or had a check after six weeks of discharge were coded as “0”. Respondents who did not know if they had a PPC visit or did not know the timing of their PPC visit were coded as missing. Within the sample, 26.7% of FTMs reported having a PPC visit within six weeks of hospital discharge.

Postnatal care within six weeks of facility discharge: This variable was based on FTMs’ answers to the questions “Did any health care provider, community health worker, or traditional birth attendant check on [CHILD’S NAME]’s health in the two months after you left [the facility]?” and “How many hours, days or weeks after the birth of [CHILD’S NAME] did that check take place?” This criterion also excluded all FTMs who did not have an institutional delivery. The timing of the PNC visit after facility discharge was recorded as hours, days, or weeks. Timely PNC visits were coded as “1” if the FTM reported her baby having a PNC visit after facility discharge within six weeks. This definition excludes postnatal checks while at the facility for delivery as 94.7% of the sample reported that their neonate was examined before hospital discharge.

**Figure A2:**
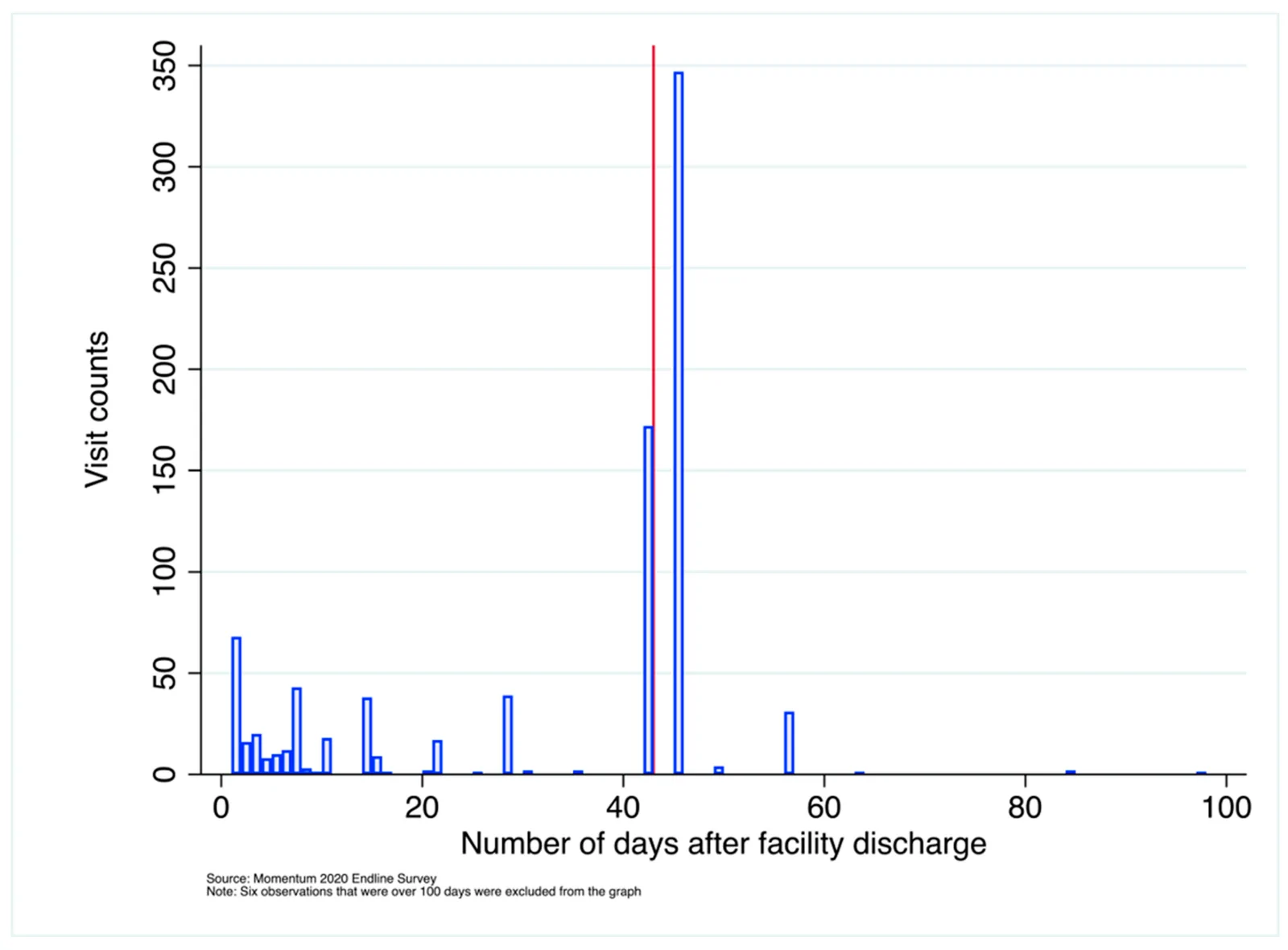
Number of postnatal care visits per day after facility discharge among FTMs aged 15–24 years old, Kinshasa, Democratic Republic of Congo. Red line denotes 42 days. The figure was created by the authors.

Like the timing of FTMs’ PPC visits, the reported duration between facility discharge and the first PNC visit heaped at 45 days. The magnitude of the possible rounding error was larger, with 39.3% of respondents reporting a PNC visit at 45 days (Appendix B Figure A2). Based on the same considerations as above, FTMs who reported that their neonate had a PNC visit at 45 days were counted as having a timely PNC visit and coded as “1”. All respondents who reported that their baby did not have a PNC after facility discharge or had a check after six weeks of discharge were coded as “0”. Respondents who did not know if their baby had a PNC visit or did not know the timing of the visit were coded as missing. Based on this definition, 46.5% of FTMs reported that their neonate had a PNC visit within six weeks of hospital discharge.

Postpartum or postnatal care within six weeks of facility discharge: A total of 365 FTMs reported that both they and their neonate had a postpartum and postnatal visit within six weeks of facility discharge and nearly two-thirds (231) reported having their PPC visit on the same day as their neonates’ PNC visit. Of the 231 FTMs who reported having a PPC check on the same day as their neonates’ PNC visit, 91.8% reported being examined by the same type of provider and 93.4% reported that the exams occurred at the same location. Respondents were not asked about the quality of care or components of the PPC or PNC visits, so it was not possible to determine if the checks were comprehensive for either the mother or the neonate or both. To account for this uncertainty, a combined outcome variable was included. A PPC or a PNC visit within six weeks of facility discharge was coded as “1”, and “0” otherwise. While the combined outcome addresses possible uncertainty in FTMs’ responses, it also obscures any differentiation between PPC and PNC care seeking.

FTM’s autonomy: FTMs’ autonomy was measured using two dimensions: decision-making and intent to engage in a specific health behavior.

Decision-making: Women’s decision-making autonomy was measured through an additive index (Cronbach’s alpha of 0.72) created using FTMs’ responses to eight questions about maternal and neonatal health decision-making in the endline survey (Appendix C). A ninth question about where to seek treatment for danger-signs in mother or newborn was excluded from the index as its factor loading was below the 0.3 cutoff. All measures of decision-making had four responses: (1) FTM only, (2) male partner only, (3) jointly, and (4) “other”. Responses of “FTM only” were coded as “2”, responses of “jointly” were coded as “1”, and responses of “male partner only” and “other” were coded as “0”. As the Momentum intervention advocated for male partner involvement and communication, the “joint” response was included. The index ranged from 0–16. Factor analysis yielded a one-factor solution. The first factor had an eigenvalue of 1.99 and accounted for 96% of the variance. The Kaiser–Meyer–Olkin (KMO) coefficient was 0.73. The index was divided into three categories for ease of interpretation.

Intent: Intent was defined using FTMs’ responses to three questions asking whether they would perform a specific action even if those important to them did not want them to. The specific actions were using postpartum contraception within six weeks of delivery, practicing Kangaroo Mother Care (KMC) if the FTM had a low birthweight or preterm baby, and practicing exclusive breastfeeding. For using postpartum contraception, FTMs were asked to mention five people most important to them and then asked if they would still use a method of contraception six weeks following childbirth even if each person mentioned did not want them to. All FTMs who said they would still use a contraceptive method if three or more of the referents did not want them to were coded as “1” and all others were coded as “0”. For practicing KMC and exclusive breastfeeding, FTMs were only asked if they would practice the method if those most important to them did not want them to; those who said yes were coded as “1” and those who said no were coded as “0”. Each question was included separately in the analysis because an additive index of the three variables had a Cronbach’s alpha of 0.38, indicating that covariance was low and the index was not reliable.

To address issues of temporality, data on intent to use postpartum family planning was from the 2018 baseline survey for Momentum. These responses are closer to the six-week period for postpartum care than those in the endline survey. The variables for intent to practice KMC and exclusive breastfeed were from the 2020 endline survey because they were not asked in the baseline survey.

Male partner involvement: Male partner involvement was measured in two ways: involvement during the antenatal period and delivery and participation in childcare.

Antenatal and delivery: Male partner involvement during the antenatal period and delivery was measured using an additive index (Cronbach’s alpha of 0.82). The index was created from FMTs’ responses to 12 questions in the endline survey about their male partners’ participation in different activities (Appendix C) during the antenatal period and during delivery. Responses of “yes” were coded as “1” and “no” responses were coded as “0” with a range of 0–12. Factor analysis showed a one factor solution with an eigenvalue of 3.55 which accounted for 93% of variance. The KMO coefficient was 0.87 and the index was divided into three terciles (“low”, “medium”, and “high”) for ease of interpretation.

Childcare: Male partner participation in childcare was an additive index (Cronbach’s alpha of 0.86) created from FTMs’ responses to eight questions about their male partner’s involvement in childcare activities (Appendix C) in the endline survey. Responses of “great deal” and “a bit” were coded as “1”, and response of “not at all” were coded as “0”. The range of the index was 0–8. Factor analysis found a one factor solution with an eigenvector value of 3.58 that accounted for 96% of the variance. The KMO coefficient was 0.87 and, like the previous additive indices, the index was divided into three categories for ease of interpretation for program managers and policy makers.

The decision-making autonomy index, the ANC and delivery male partner involvement index, and the childcare male partner involvement index were all additive to make results easier to understand by program managers and policy makers. As the indices represent composite measures and not inherently continuous variables, they were categorized into three groups to make the main effects and interaction terms more interpretable. Separate analyses treating the indices as continuous rather than categorical yielded similar results.

Additional FTM-related covariates were age, socioeconomic status, education, employment, relationship status, health zone of residence, media exposure, and intention status of the pregnancy. Age was measured as a binary variable, 15–19 years (reference) versus 20–24 years. FTMs’ education was defined using two categories, “none/primary/secondary incomplete” (reference) or “secondary complete/higher”. Employment was measured by asking if the FTM had worked, aside from housework, in the 12 months preceding the baseline survey (yes or no), and residence measured whether the FTM lived in an intervention health zone (1) or a comparison health zone (0) at baseline. Relationship status was defined as in-union or not. FTMs who were married or living with a male partner were coded as “1” or and all other responses were coded as “0”. Media exposure measured whether the FTM watched television at least once a week (1) or less often (0).

Unintended pregnancy was measured using FTMs’ responses to the question “When you got pregnant, did you want to get pregnant at that time?” FTMs who said “yes” were coded as “1” and those who said “no” were coded as “0”. Socioeconomic status was an index created using principal component analysis based on assessments of the floor, wall, and roofing materials of the respondent’s dwelling unit, drinking water, toilet facilities, fuel, electricity, and ownership of items (Conbach’s alpha of 0.64). The index was divided into terciles: low, medium, and high.

### 2.4. Data Analysis

Descriptive statistics were presented as percentages for both FTMs included and excluded from the sample. Bivariate analysis used unadjusted logistic regression to test the association between the outcome variables, explanatory variables, and socio-demographic variables. As the sample was purposive, the data were not weighted.

This paper estimated two multivariable logistic regression models for each outcome. The first regression model (Model 1) included all variables of interest, and the second model (Model 2) included the same variables and two interaction terms: (1) male involvement in maternal care and in-union status, and (2) male involvement in childcare and in-union status. Logistic regression results are presented in the form of odds ratios (ORs) and 95% confidence intervals (CIs).

Multicollinearity was assessed using variance inflation factors (VIFs), once for all independent variables and postnatal and postpartum variables, and once for all independent variables and the combined outcome. For the PPC and PNC outcomes, the highest VIF value was 1.34 with a mean VIF value of 1.17, indicating multicollinearity was not of concern [50]. A correlation matrix between all the variables showed low correlation between the variables, with the highest correlation coefficient being 0.44 between employment status and age group. For the combined dependent variable, the highest VIF value was 1.34 and the mean VIF value was 1.16. The highest correlation coefficient was 0.44 between women’s education and women’s age. Statistical analysis was performed using Stata software version 16 (StataCorp, College Station, TX, USA) [51].

A total of 798 FTMs were excluded from analysis; 504 FTMs were lost-to-follow-up, nine were dropped due to missing demographic data, 47 did not have a facility delivery, 70 were not asked about the timing of a postpartum visit after facility delivery due to having a stillbirth, and 168 were dropped for missing data on receiving a timely PPC or PNC visit. A Pearson’s chi-square test between those included and excluded from analysis found that there were statistically significant differences in household wealth, health zone residence, and pregnancy intentions between included and excluded cases. Compared to FTMs who were included in the analysis, more of those who were excluded lived in the lowest wealth households (41% vs. 33%) and had planned pregnancies (22% vs. 17%). For the health zone of residence, the largest difference was in Kisagani, followed by Masina1 and Ndjili. Mean age, employment, educational attainment, and television exposure were similar between the groups.

## 3. Results

### 3.1. Participants’ Characteristics

Table 1 shows the demographic characteristics of FTMs by inclusion in the analysis. The mean age of study participants was 19.9 years, with almost two-thirds unemployed in the year before the baseline survey (Table 1). Over half of respondents had no schooling, a primary education, or an incomplete secondary education. A little more than 40% of FTMs were in-union and over 80% had an unplanned pregnancy. About half of participants were 15–19 years old.

### 3.2. Bivariate Analysis

Table 2 presents bivariate analysis of all variables of interest and each outcome. Autonomy in deciding to practice KMC and high male involvement in childcare were positively associated with all outcomes. For neonates, autonomy in deciding to practice exclusive breastfeeding was also associated with a timely PNC visit and a timely PNC or PPC visit. For both the combined outcome and PNC, high male involvement in maternal care was associated with decreased odds of a timely postpartum visit. In-union status was associated with timely postpartum care for the combined outcome. The highest tercile of male involvement in childcare was positively associated with all outcomes. FTMs in the intervention health zones had higher odds of the FTM having a PPC visit within six weeks of facility discharge and lower odds of their neonate having a timely PNC visit.

### 3.3. Multivariable Analysis

Table 3 shows adjusted ORs from our multivariable logistic regression models. In Model 1 the male involvement in childcare index was positively and significantly associated with all outcomes. Compared to low involvement, FTMs who reported their partner was highly involved had increased odds of having a PPC visit (OR = 1.52; 95% CI = (1.11–2.08)), a PNC visit (OR = 1.94; 95% CI = (1.45–2.59)), or either visit (OR = 1.73; 95% CI = (1.30–2.30)). Additionally for PNC visits or either visit, the medium category of the male involvement in childcare was significant and positively associated with a timely visit (OR = 1.36; 95% CI = (1.07–1.73)) compared to the low category.

In contrast, the male involvement in maternal care index was significantly and negatively associated with all outcomes. For PPC visits, moderate involvement was associated with a 25% decrease (OR = 0.75; 95% CI = (0.57–0.99)) in the odds of a timely visit compared to low involvement. For PNC visits, FTMs with male partners who were highly involved in the antenatal and delivery periods had a 41% decrease in the odds (OR = 0.59; 95% CI = (0.45–0.78)) of a timely visit compared to those who reported low male involvement. For the combined outcome, FTMs who reported that their male partners were highly involved had a 29% decrease in the odds (OR = 0.71; 95% CI = (0.54–0.93)) of a timely PPC or PNC visit compared to FTMs who reported low male involvement in the maternal care index. Newborns of FTMs who were in union had decreased odds (OR = 0.80 95% CI = (0.65–0.98)) of a timely PNC visit compared to those of FTMs who were not. FTMs who were in union had decreased odds (OR = 0.76; 95% CI = (0.61–0.93)) of a timely PPC or PNC compared to those who were not. Finally, the odds of a timely PPC visit were 50% higher in intervention health zones compared to comparison health zones. The odds of a timely PNC visit were 25% lower in comparison health zones compared to intervention health zones.

In Model 2 only, the interactions between the male involvement indices and the PPC outcome were statistically significant. In-union FTMs who had a male partner with medium involvement in maternal care had nearly 50% decreased odds (OR = 0.56; 95% CI = (0.32–0.98)) of having a timely care visit compared to FTMs who were not in-union with a male parter who had moderate or low involvement. In-union FTMs with a partner moderately involved in childcare had an 89% increase in the odds (OR = 1.89; 95% CI = (1.06–3.39)) of having a timely PPC visit compared to non-in-union FTMs with similarly involved or low-involvement partners.

A sensitivity analysis was conducted using the WHO-recommended 42-day definition (Appendix A Table A4). This analysis yielded similar results, though several associations were no longer statistically significant. Under the WHO cutoff, 21.4% of FTMs had a timely PPC visit, 27.3% of neonates had a timely PNC visit, and 36.2% of respondents either had a timely PPC or PNC visit. KMC autonomy remained positively associated with all outcomes. The male involvement in childcare index was no longer significantly associated with any outcomes, while high male involvement in maternal care remained negatively associated with PNC utilization (OR = 0.60; 95% CI = (0.44–0.82). Although the autonomy index estimates changed direction, its association with all outcomes remained non-significant. Chi-square tests showed that FTMs reporting PPC and PNC at 45 days differed significantly on the autonomy index, education, and employment from those reporting PPC and PNC using the 42-day cutoff. FTMs reporting care within 45 days of facility discharge had a higher percentage of respondents in the lowest autonomy category (PPC: 33.9% vs. 60.2; PNC: 34.6% vs. 43.8%). Respondents who reported a 45-day visit were also significantly less educated for the PPC outcome (54.4% vs. 66.7%) and fewer had worked last year for the PNC outcome (59.1% vs. 66.1%).

## 4. Discussion

The models found conflicting results regarding male partner participation. While male partner involvement in childcare was positively associated with all outcomes, male partner involvement in maternal care was negatively associated with all outcomes. These findings are in contrast with other studies which found that male involvement in maternal care was positively associated with improved health outcomes, including increased postpartum care utilization [21,52,53].

The negative association between the male involvement in maternal care index and postnatal care-seeking was robust and remained after disaggregating by age and in-union status (Appendix A Table A1, Table A2 and Table A3) and dividing the index into separate components. When testing items individually, male attendance at delivery was negatively associated with postnatal care-seeking, which does not match other findings on male partner delivery attendance [19]. One study in England found that when male partners became anxious or stressed in the delivery room it worsened labor difficulty, but there was no evidence it impacted health utilization [54].

It is possible that male partner birth attendance was a signal of control rather than support [55]. Another study using Momentum data found that among older male participants, those who were perpetrators of emotional violence were more involved in ANC and birth preparedness than non-perpetrators [56]. Qualitative interviews with study participants found that some FTMs inflated the cost of prenatal care to their partners in order to receive more money when being reimbursed by their partners [57]. It may be that partners who accompany FTMs to prenatal appointments find out the real cost of services and are reluctant to give the FTM more money for postnatal services. Other studies have also reported negative consequences of male involvement. A study in Turkey found that after men received education on pregnancy nutrition, exclusive breastfeeding, and support behaviors, some female partners felt infantilized by partners using their new knowledge to tell them what to eat [26]. Research in Ghana found that newborn care was an area in which women held significant power and autonomy in an otherwise patriarchal society and that male involvement could decrease women’s already limited power [58]. There may be underlying social and household dynamics that are not fully measured by the index, so caution is warranted in interpreting a direct negative influence between male partner involvement in maternal care and postpartum or postnatal care seeking. Additional research into male partner involvement in maternal care among adolescent and young FTMs is needed to better understand this finding.

Disaggregation by age and in-union status showed that the negative association between the male involvement in maternal care index and PPC visits was only significant for in-union 20–24-year-old FTMs, but was significant for both the PNC and the combined outcomes for all FTMs except 15–19-year-olds who were not in union (Appendix A Table A1, Table A2 and Table A3). These findings could indicate that older in-union FTMs may need additional support to access PPC and that FTMs across age and union status categories possibly need further support in accessing timely PNC for their newborns. However, this cannot be ascertained as the study is cross-sectional.

Male involvement in childcare was positively associated with timely postpartum and postnatal care, matching previous findings linking male involvement to improved health in children, mostly in high-income countries [59,60,61]. This finding is important as most research on male involvement in childcare in LMICs has focused on how to increase fatherhood involvement rather than the impact of fatherhood on maternal and child health outcomes. Additionally, most parenting interventions to improve early childhood development have focused only on the mother while excluding the father [62]. While it is not possible to definitively link male involvement in childcare to the postpartum period based on this study’s findings, the results indicate that there may be an underlying factor in which greater male involvement in childcare is closely connected to greater support of for partners utilization of healthcare during the postpartum period. Encouraging more male involvement in early childcare presents a possible strategy for improved maternal and neonatal health outcomes in the DRC, but research establishing causality is needed.

Analysis disaggregating Model 1 by in-union status and age (Appendix A Table A1, Table A2 and Table A3) found that male involvement in childcare was positively associated with all outcomes for all groups except PPC and PPC/PNC for FTMs aged 15–19 years who were not in-union and PNC for their counterparts who were not, meaning that younger FTMs may need additional support.

The conflicting results of the two male involvement indices may reveal a discrepancy between the social norms underlying the two measurements in the DRC. It is possible that men who were involved in maternal care did so to control their partners, matching established social norms of men as the primary decision-makers [23], while fathers who engage in childcare act against patriarchal ideals and are therefore more likely to support care-seeking for their partner and newborn. Additional research into men’s roles in maternal and newborn health in the DRC is needed so programs know how to effectively engage with gender norms to encourage male partners’ support of women’s healthcare utilization.

The results from both models showed that neither dimension of autonomy, decision-making, or intent to engage in specific health behaviors was associated with timely postpartum or postnatal care among FTMs and their newborns. Although autonomy in deciding to practice KMC was positively associated with all outcomes, a single measure of autonomy is not sufficient to make firm conclusions about the relationship between women’s autonomy and postpartum care-seeking. These findings may be due to how autonomy was measured in the study rather than the absence of a relationship. Results from previous studies on the association between women’s autonomy and reproductive health care outcomes in the DRC have varied, depending on how autonomy was defined and the outcome used. Autonomy is a multifaceted concept [63], and the null findings only indicate that there is no association between the dimensions of autonomy measured and the outcomes.

It may be that decision-making autonomy was not a good measure of autonomy among adolescent and young FTMs. A study in Nepal found that husbands were the most influential decision-maker for adolescent and young women when deciding to access ANC services, compared to the woman being the decision-maker among older women [64]. While it may be that younger women are more likely to have their autonomy restricted by their partners, they also could feel less qualified to make decisions, especially as first-time mothers, and their partner as the decision-maker is their desired outcome. Formative research is needed to better understand gender roles and autonomy within the context of the DRC and adolescent and young first-time mothers to develop more appropriate measures. The robust positive association between the intent to practice KMC and postpartum and postnatal care seeking could offer insights into how autonomy could be conceptualized for this population in future studies.

Support for effect modification by in-union status on the association between male partner involvement and care-seeking behavior was limited. In-union status was found to moderate the relationship between the male involvement indices and PPC. These findings show that there may be differences in support during pregnancy and after childbirth within established partnerships, but findings need to be confirmed by future studies.

The sensitivity analysis using the WHO 42-day cutoff found the association between KMC autonomy and all outcomes and the male maternal care index and PNC to be robust. Though the male involvement in childcare index was no longer significantly associated with any outcome, the directionality of the estimates remained the same. The reclassification of a substantial number of visits (18.9% for PPC and 39.3% for PNC) in each dependent variable may account for some of the loss in significance as there were fewer events. Additionally, the two groups of respondents were found to differ significantly on some variables. Overall, the sensitivity analysis showed that the study findings are supported but sensitive to the operational definition of timely care.

Postnatal care-seeking was negatively associated with being in a Momentum intervention health zone, while maternal health had a positive association. Newborns in the comparison health zone of Bumbu had statistically significant higher odds of having a timely postnatal check compared to all other health zones (Appendix A Table A1, Table A2 and Table A3). This increase may be due to the “Access to Health Services in Kinshasa” (ASSK) project which was implemented in the Bumbu health zone from 2018–2023. The ASSK project aimed to improve the quality and availability of sexual and reproductive health services and the utilization of these services by women, children, and adolescents [65]. Additionally, it may be due to the Momentum intervention emphasizing maternal care more than newborn care. The intervention areas may have had better maternal health services and weaker newborn care services. Both intervention and comparison areas were selected from health zones in which another project was involved in improving the quality of care in high-volume maternity hospitals. Health facility data on the quality of newborn care were not assessed at the time of health zone selection and there may have been uneven maternal–newborn health integration in the health zones of study and lower quality of newborn health services in intervention than in comparison health zones, creating an imbalance. These reasons are a matter of conjecture, and further research is needed to clarify the nuances in health zone differentials in Momentum’s impact on newborn care.

Finally, these findings demonstrate a need to better understand demographic factors associated with postpartum and postnatal care utilization in the DRC among young FTMs. Characteristics associated with PPC and PNC utilization both in sub-Saharan Africa and the DRC, such as high socioeconomic status, maternal education, wealth, four or more ANC visits, institutional delivery, media exposure, and employment status [8,66,67] were not significant in this study population (~90% of the study sample had four or more ANC visits and delivered at a health facility).

This study has some limitations that should be considered when evaluating the results. First, the study was cross-sectional; thus, causality cannot be established. This study was non-experimental and while comparison zones were selected based on similar demographic characteristics, there is likely some uncontrolled confounding based on non-randomized selection. This study was unable to account for some key determinants of postpartum care such as healthcare costs, quality of care, transportation access, and family support and there may be some residual confounding due to these unmeasured variables.

The endline survey was conducted 18 months after delivery and there may be recall bias about the timing and receiving of a postpartum or postnatal check. The questions focused on the period immediately following birth which may be an especially stressful time for mothers and the experience of becoming a mother may have exacerbated poor recall. There may also be recall bias concerning questions about male involvement during pregnancy and delivery as almost two years had elapsed between the survey and the time in question.

There is a possibility of sampling bias, as some FTMs were recruited at ANC visits, indicating that these respondents had access to and utilized maternal health care and thus may be more likely to have a PPC or PNC visit than FTMs recruited through the community. However, the number of women recruited at ANC visits was under 50 and ANC utilization in the sample was 95%, indicating that both those recruited at clinics and in the community were able and willing to use maternal care.

FTMs who were lost to follow up or excluded based on missing outcome data differed significantly from included FTMs by socioeconomic status, health zone, and pregnancy intention. Excluded FTMs were poorer and this could have resulted in an overestimation of the outcome variables, as high socioeconomic status is associated with increased care seeking [8]. Additionally, access to health facilities may vary by health zone meaning that differential exclusion by health zone could have influenced estimates. These differences between the included and excluded groups and the purposive nature of the sample may indicate that the findings are less generalizable to other FTMs in Kinshasa.

Another limitation of this study was that knowledge of postpartum and postnatal care was not assessed. Low knowledge of postpartum care is associated with low utilization [68], and it is not clear if FTMs knew they should seek routine postpartum and postnatal care. Over 90% of FTMs reported that both they and their baby had a health check before facility discharge, and it is possible they assumed this check was sufficient. It is not known if healthcare providers communicated to the FTMs about postpartum or postnatal care. The Momentum project did teach FTMs and their male partners about obstetric and newborn danger signs, and 97% of those who experienced a postpartum complication sought care at a facility, but routine postpartum care was not emphasized. Analysis controlling for care-seeking for obstetric or newborn emergencies did not find significance.

Except for PPFP autonomy, all autonomy variables were measured in the endline survey, which was administered about 18 months after delivery. Autonomy can change over time [46] so it is possible that FTMs’ reported autonomy in the endline survey was not the same as their autonomy during the postpartum period. For the decision-making index, most questions were related specifically to the antenatal, delivery, and after-delivery time periods (though not specific to the six-week postpartum period) (Appendix C). The breastfeeding and KMC autonomy variables may be more susceptible to issues of temporality as FTMs were asked about their intention to perform a behavior in the future. Estimates are based on the association between FTMs’ autonomy at endline and postpartum care seeking rather than their autonomy during the postpartum period.

Finally, the timing of fathers’ involvement in childcare cannot be specifically attributed to the postpartum period. Male childcare involvement was only measured in the endline survey. Although the questions about male involvement in childcare specifically ask about participation “after the baby was born”, the postpartum period was not explicitly identified. All activities FTMs were asked about (Appendix C) could apply to a child at any time during the first 18 months and so responses to male involvement could include fathers who were only involved in childcare after the postpartum period. Analysis including male partner responses to willingness to perform certain childcare activities in the baseline survey, as the baseline survey was administered closer to the postpartum period, found no significant association between willingness to engage in childcare and any outcome.

## 5. Conclusions

This study examined the associations between male partner involvement, women’s autonomy, and timely postpartum and postnatal care utilization among young first-time mothers in Kinshasa, DRC. Male partner involvement in childcare was positively associated with timely PPC and PNC, while male partner involvement in maternal care was negatively associated with study outcomes. Women’s autonomy was not consistently associated with timely PPC or PNC utilization. Almost no demographic characteristics were associated with any outcome. These findings highlight the importance of understanding young FTMs as a distinct population. The lack of association between women’s autonomy and postpartum care use suggests that measures of autonomy may not be adequately contextualized for the study population. Appropriate measures of women’s autonomy should be developed and validated before interventions are designed around the construct. Similarly, additional research is needed to confirm the male partner engagement findings, establish causality, and refine measures of male involvement to distinguish between supportive partner support and male control within the context of the DRC.

## Figures and Tables

**Table 1:** Percent distribution of first-time mothers (FTMs) aged 15–24 years by inclusion status, according to background characteristics, Kinshasa Democratic Republic of the Congo 2018.

Background Characteristics	FTMs Included		FTMs Excluded		Sig.
	n	%	n	%	
Age group					
15–19	855	48.9	317	46.6	
20–24	895	51.1	364	53.4	
FTM’s highest level of schooling					
None/primary/secondary incomplete	1026	58.6	422	62.0	
Secondary complete/higher	724	41.4	259	38.0	
Household wealth					***
Low	579	33.1	272	40.7	
Medium	592	33.8	223	33.3	
High	579	33.1	174	26.0	
In-union					
No	750	42.9	263	38.6	
Yes	1000	57.1	418	61.4	
Worked last year					
No	1120	64.0	433	63.6	
Yes	630	36.0	248	36.4	
Watches TV at least once a week					
No	645	36.9	275	40.4	
Yes	1105	63.1	406	59.6	
Unintended pregnancy					**
No	300	17.1	151	22.2	
Yes	1450	82.9	530	77.8	
Health zone of residence					***
Bumbu	217	12.4	85	12.5	
Kingasani	396	22.6	207	30.4	
Lemba	232	13.3	103	15.1	
Masina1	378	21.6	122	17.9	
Matete	215	12.3	66	9.7	
Ndjili	312	17.8	98	14.4	
N	1750		681		

** *p* < 0.01, *** *p* < 0.001. Source: Momentum 2018 Female Baseline Survey.

**Table 2:** Bivariate analysis of first-time mothers aged 15–24 years who report having a timely postpartum care or postnatal care visit and explanatory variables.

Variables	Postpartum Visit Within Six Weeks		Postnatal Visit Within Six Weeks		Postpartum/Postnatal Visit Within Six Weeks	
	Crude OR	95% CI	Crude OR	95% CI	Crude OR	95%CI
Decision-making autonomy						
Low	-		-		-	
Medium	0.90	(0.70–1.15)	0.88	(0.70–1.09)	0.87	(0.70–1.08)
High	0.89	(0.68–1.17)	0.97	(0.77–1.24)	0.95	(0.75–1.20)
Autonomy: Postpartum contraception						
No	-		-		-	
Yes	1.08	(0.87–1.34)	1.18	(0.98–1.43)	1.17	(0.96–1.41)
Autonomy: Kangaroo Mother Care						
No	-		-		-	
Yes	1.53 **	(1.20–1.96)	1.68 ***	(1.36–2.08)	1.82 ***	(1.48–2.25)
Autonomy: Breastfeeding						
No	-		-		-	
Yes	1.09	(0.88–1.36)	1.27 *	(1.05–1.54)	1.38 **	(1.14–1.67)
Male involvement in maternal care						
Low	-		-		-	
Medium	0.80	(0.62–1.04)	0.94	(0.75–1.17)	0.98	(0.78–1.22)
High	1.06	(0.82–1.38)	0.66 **	(0.52–84)	0.77 *	(0.61–0.98)
Male involvement in childcare						
Low	-		-		-	
Medium	1.12	(0.87–1.43)	1.10	(0.89–1.37)	1.14	(0.92–1.41)
High	1.41 *	(1.06–1.86)	1.35 *	(1.05–1.74)	1.29 *	(1.00–1.66)
Unintended pregnancy						
No	-		-		-	
Yes	0.95	(0.72–1.27)	0.86	(0.67–1.10)	0.84	(0.65–1.07)
Age group						
15–19	-		-		-	
20–24	1.06	(0.85–1.30)	1.02	(0.85–1.23)	1.04	(0.86–1.26)
In-union						
No	-		-		-	
Yes	0.92	(0.74–1.14)	0.84	(0.69–1.01)	0.81 *	(0.67–0.98)
FTM’s highest level of schooling						
None/primary/secondary incomplete	-		-		-	
Secondary complete or higher	1.11	(0.89–1.37)	1.04	(0.86–1.25)	1.12	(0.93–1.36)
Household wealth						
Low	-		-		-	
Medium	0.90	(0.69–1.17)	0.98	(0.78–1.23)	0.89	(0.71–1.12)
High	1.08	(0.84–1.40)	1.05	(0.84–1.33)	1.00	(0.80–1.26)
Worked last year						
No	-		-		-	
Yes	1.14	(0.92–1.42)	1.18	(0.97–1.43)	1.19	(0.98–1.44)
Watches TV at least once a week						
No	-		-		-	
Yes	1.06	(0.85–1.32)	0.96	(0.79–1.16)	0.99	(0.81–1.20)
Health zone						
Comparison	-		-		-	
Intervention	1.52 ***	(1.23–1.88)	0.75 **	(0.62–0.90)	0.87	(0.72–1.05)
N	1750		1750		1750	

* *p* < 0.05, ** *p* < 0.01, *** *p* < 0.001. Source: Momentum 2018 FTM Baseline Survey and 2020 FTM Endline Survey.

**Table 3:** Multivariable logistic regression model of timely postpartum care visit among first-time mothers aged 15–24 years, Kinshasa, Democratic Republic of the Congo 2020.

Variables	Model 1						Model 2					
	Postpartum Care		Postnatal Care		Postpartum/Postnatal Care		Postpartum Care		Postnatal Care		Postpartum/Postnatal Care	
	Adjusted Odds Ratio	95% CI	Adjusted Odds Ratio	95% CI	Adjusted Odds Ratio	95% CI	Adjusted Odds Ratio	95% CI	Adjusted Odds Ratio	95% CI	Adjusted Odds Ratio	95% CI
Decision-making autonomy												
Low	-		-		-		-		-		-	
Medium	0.90	(0.70–1.16)	0.87	(0.69–1.09)	0.85	(0.68–1.07)	0.90	(0.70–1.15)	0.87	(0.69–1.09)	0.85	(0.68–1.06)
High	0.91	(0.69–1.20)	0.95	(0.74–1.22)	0.94	(0.73–1.20)	0.90	(0.68–1.19)	0.94	(0.73–1.21)	0.92	(0.72–1.19)
Autonomy: Postpartum contraception												
No	-		-		-		-		-		-	
Yes	1.10	(0.88–1.37)	1.13	(0.93–1.38)	1.13	(0.93–1.37)	1.12	(0.90–1.40)	1.14	(0.94–1.39)	1.14	(0.94–1.39)
Autonomy: Kangaroo Mother Care												
No	-		-		-		-		-		-	
Yes	1.55 **	(1.18–2.03)	1.60 ***	(1.26–2.02)	1.69 ***	(1.34–2.13)	1.56 **	(1.19–2.05)	1.60 ***	(1.26–2.02)	1.70 ***	(1.35–2.14)
Autonomy: Exclusive Breastfeeding												
No	-		-		-		-		-		-	
Yes	0.95	(0.75–1.20)	1.11	(0.90–1.38)	1.17	(0.95–1.45)	0.95	(0.74–1.21)	1.11	(0.90–1.38)	1.17	(0.95–1.45)
Male involvement in maternal care												
Low	-		-		-		-		-		-	
Medium	0.75 *	(0.57–0.99)	0.87	(0.68–1.10)	0.92	(0.72–1.17)	0.95	(0.67–1.35)	0.97	(0.71–1.33)	1.06	(0.78–1.46)
High	0.92	(0.68–1.24)	0.59 ***	(0.45–0.78)	0.71 *	(0.54–0.93)	1.06	(0.70–1.60)	0.66 *	(0.45–0.96)	0.80	(0.55–1.16)
Male involvement in childcare												
Low	-		-		-		-		-		-	
Medium	1.22	(0.93–1.60)	1.36 *	(1.07–1.73)	1.36 *	(1.07–1.72)	0.95	(0.67–1.33)	1.21	(0.89–1.64)	1.15	(0.85–1.56)
High	1.52 *	(1.11–2.08)	1.94 ***	(1.45–2.59)	1.73 ***	(1.30–2.30)	1.50	(0.98–2.32)	2.05 **	(1.36–3.10)	1.90 **	(1.25–2.88)
Unintended pregnancy												
No	-		-		-		-		-		-	
Yes	0.93	(0.69–1.26)	0.90	(0.68–1.17)	0.86	(0.66–1.13)	0.95	(0.70–1.29)	0.90	(0.66–1.18)	0.88	(0.67–1.15)
Age group												
15–19	-		-		-		-		-		-	
20–24	1.05	(0.82–1.34)	0.89	(0.68–1.17)	0.99	(0.80–1.24)	1.04	(0.81–1.34)	0.99	(0.79–1.24)	0.99	(0.79–1.24)
In-union												
No	-		-		-		-		-		-	
Yes	0.85	(0.67–1.07)	0.80 *	(0.65–0.98)	0.76 **	(0.61–0.93)	0.83	(0.50–1.36)	0.86	(0.56–1.32)	0.82	(0.54–1.26)
FTM’s highest level of schooling												
None/primary/secondary incomplete	-		-		-		-		-		-	
Secondary complete or higher	1.07	(0.83–1.37)	1.05	(0.84–1.32)	1.14	(0.92–1.43)	1.07	(0.83–1.37)	1.06	(0.84–1.32)	1.15	(0.92–1.44)
Household wealth												
Low	-		-		-		-		-		-	
Medium	0.86	(0.65–1.14)	0.95	(0.75–1.22)	0.85	(0.66–1.09)	0.85	(0.64–1.12)	0.95	(0.74–1.21)	0.84	(0.65–1.07)
High	1.05	(0.79–1.40)	1.04	(0.81–1.35)	0.96	(0.74–1.24)	1.07	(0.80–1.43)	1.05	(0.81–1.36)	0.97	(0.75–1.25)
Worked last year												
No	-		-		-		-		-		-	
Yes	1.14	(0.91–1.43)	1.20	(0.97–1.47)	1.20	(0.98–1.48)	1.15	(0.92–1.45)	1.20	(0.98–1.48)	1.22	(0.99–1.49)
Watches TV at least once a week												
No	-		-		-		-		-		-	
Yes	1.04	(0.82–1.33)	0.94	(0.76–1.16)	0.99	(0.80–1.22)	1.04	(0.82–1.33)	0.94	(0.76–1.16)	0.99	(0.80–1.22)
Intervention health zone												
Comparison	-		-		-		-		-		-	
Intervention	1.50 ***	(1.20–1.87)	0.75 **	(0.62–0.91)	0.87	(0.71–1.05)	1.51 ***	(1.21–1.88)	0.75 **	(0.62–0.92)	0.87	(0.72–1.06)
Interaction terms												
Male involvement in maternal care:In-union												
Low:In-union							-		-		-	
Medium:In-union							0.56 *	(0.32–0.98)	0.75	(0.46–1.22)	0.70	(0.43–1.14)
High:In-union							0.69	(0.38–1.25)	0.77	(0.45–1.33)	0.73	(0.42–1.25)
Male involvement in childcare:In-union												
Low:In-union							-		-		-	
Medium:In-union							1.89 *	(1.06–3.39)	1.23	(0.79–2.14)	1.44	(0.88–2.36)
High:In-union							1.23	(0.63–2.38)	0.97	(0.53–1.75)	0.93	(0.52–1.69)
N							1750		1750		1750	

* *p* < 0.05, ** *p* < 0.01, *** *p* < 0.001. Source: Momentum 2018 Female Baseline Survey and 2020 Female Endline Survey.

## Data Availability

The original contributions presented in this study are included in the article/Supplementary Material File S1. Further inquiries can be directed to the corresponding author.

## Supplementary Materials

The following supporting information can be downloaded at: https://www.mdpi.com/article/10.3390/ijerph23081061/s1, File S1: ppc_data.csv.

## Abbreviations

The following abbreviations are used in this manuscript:

DRC	Democratic Republic of Congo
FTM	First-time mother
WHO	World Health Organization
LMIC	Low- and middle-income country
PPC	Postpartum care
PNC	Postnatal care
ANC	Antenatal care
DHS	Demographic and Health Survey
VIF	Variance inflation factor

## Appendix A

**Table A1:** Multivariable logistic regression model of timely postpartum care visit among first-time mothers aged 15–24 years, Kinshasa, DRC 2020 by age and relationship status.

Variables	Model 1							
	Postpartum Care							
	15–19 Years				20–24 Years			
	In-Union		Not In-Union		In-Union		Not On-Union	
	Adjusted Odds Ratio	95% CI	Adjusted Odds Ratio	95% CI	Adjusted Odds Ratio	95% CI	Adjusted Odds Ratio	95% CI
Decision-making autonomy								
Low	-							
Medium	0.98	(0.51–1.88)	0.81	(0.51–1.30)	0.75	(0.47–1.20)	1.16	(0.65–2.07)
High	1.10	(0.53–2.30)	0.98	(0.60–1.60)	0.56	(0.31–1.02)	1.01	(0.55–1.85)
Autonomy: Postpartum contraception								
No	-		-		-		-	
Yes	0.77	(0.43–1.36)	0.76	(0.51–1.15)	1.46	(0.94–2.28)	0.74	(0.46–1.22)
Autonomy: Kangaroo Mother Care								
No	-		-		-		-	
Yes	1.59	(0.81–3.14)	1.42	(0.86–2.35)	1.57	(0.90–2.74)	1.20	(0.66–2.18)
Autonomy: Breastfeeding								
No	-		-		-		-	
Yes	0.76	(0.40–1.43)	1.32	(0.85–2.05)	0.77	(0.47–1.26)	0.83	(0.50–1.37)
Male involvement in maternal care								
Low	-		-		-		-	
Medium	0.58	(0.28–1.22)	1.14	(0.71–1.83)	0.42 **	(0.24–0.76)	0.85	(0.48–1.51)
High	0.79	(0.37–1.68)	1.03	(0.59–1.80)	0.49 *	(0.27–0.91)	1.15	(0.59–2.26)
Male involvement in childcare								
Low	-		-		-		-	
Medium	1.76	(0.80–3.88)	0.96	(0.61–1.52)	1.96 *	(1.04–3.67)	0.89	(0.51–1.57)
High	2.44 *	(1.01–5.92)	1.23	(0.66–2.31)	1.92	(0.98–3.77)	1.98 *	(1.02–3.85)
Unintended pregnancy								
No	-		-		-		-	
Yes	1.61	(0.68–3.79)	0.78	(0.31–1.98)	1.05	(0.66–1.66)	0.87	(0.44–1.74)
FTM’s highest level of schooling								
None/primary/secondary incomplete	-		-		-		-	
Secondary complete or higher	1.69	(0.87–3.26)	0.86	(0.51–1.45)	1.61 *	(1.01–2.56)	0.66	(0.40–1.09)
Household wealth								
Low					-		-	
Medium	0.90	(0.47–1.74)	0.79	(0.49–1.27)	0.78	(0.45–1.34)	1.01	(0.52–1.95)
High	1.04	(0.49–2.18)	0.92	(0.56–1.51)	0.85	(0.48–1.48)	1.83	(0.96–3.48)
Worked last year								
No	-		-		-		-	
Yes	1.11	(0.59–2.10)	1.11	(0.72–1.73)	1.17	(0.76–1.79)	1.18	(0.72–1.93)
Watches TV at least once a week								
No	-		-		-		-	
Yes	0.60	(0.32–1.13)	1.26	(0.82–1.95)	0.98	(0.61–1.56)	1.38	(0.79–2.41)
Intervention health zone								
Bembu	-		-		-		-	
Kingasani	0.69	(0.29–1.64)	1.60	(0.82–3.11)	2.52 *	(1.09–5.85)	0.64	(0.27–1.50)
Lemba	0.29 *	(0.10–0.86)	1.34	(0.64–2.79)	1.89	(0.74–4.82)	0.82	(0.32–2.05)
Masina1	0.16 **	(0.05–0.51)	0.47	(0.21–1.04)	1.29	(0.55–3.05)	0.33 *	(0.14–0.81)
Matete	0.78	(0.26–2.35)	0.83	(0.38–1.83)	2.99 *	(1.22–7.31)	0.60	(0.25–1.48)
Ndjili	0.33	(0.11–0.96)	1.05	(0.53–2.10)	2.60 *	(1.08–6.28)	0.55	(0.24–1.26)
N	315		540		497		398	

* *p* < 0.05, ** *p* < 0.01. Source: Momentum 2018 Baseline Survey and 2020 Endline Survey.

**Table A2:** Multivariable logistic regression model of timely postnatal care visit among first-time mothers aged 15–24 years, Kinshasa, DRC 2020 by age and relationship status.

Variables	Model 1							
	Postnatal Care							
	15–19 Years				20–24 Years			
	In-Union		Not In-Union		In-Union		Not On-Union	
	Adjusted Odds Ratio	95% CI	Adjusted Odds Ratio	95% CI	Adjusted Odds Ratio	95% CI	Adjusted Odds Ratio	95% CI
Decision-making autonomy								
Low	-		-		-		-	
Medium	1.13	(0.64–1.99)	0.71	(0.46–1.09)	0.86	(0.56–1.32)	0.77	(0.46–1.30)
High	1.00	(0.52–1.92)	1.05	(0.66–1.65)	0.79	(0.47–1.31)	0.68	(0.39–1.16)
Autonomy: Postpartum contraception								
No	-		-		-		-	
Yes	0.64	(0.38–1.05)	1.02	(0.70–1.48)	1.43	(0.97–2.12)	0.84	(0.54–1.31)
Autonomy: Kangaroo Mother Care								
No	-		-		-		-	
Yes	1.58	(0.89–2.80)	1.75 *	(1.12–2.72)	1.70 *	(1.04–2.77)	1.27	(0.75–2.13)
Autonomy: Breastfeeding								
No	-		-		-		-	
Yes	1.08	(0.62–1.88)	0.96	(0.64–1.43)	1.09	(0.70–1.71)	1.08	(0.69–1.72)
Male involvement in maternal care								
Low	-		-		-		-	
Medium	0.66	(0.35–1.24)	1.01	(0.65–1.56)	0.70	(0.41–1.17)	0.82	(0.50–1.36)
High	0.41 *	(0.21–0.82)	0.71	(0.42–1.19)	0.39 **	(0.22–0.69)	0.42 **	(0.22–0.79)
Male involvement in childcare								
Low	-		-		-		-	
Medium	1.92	(0.99–3.73)	1.23	(0.81–1.86)	1.65	(0.97–2.80)	1.28	(0.78–2.12)
High	1.86	(0.87–3.95)	2.59 **	(1.43–4.70)	2.48 **	(1.39–4.42)	2.28 *	(1.20–4.34)
Unintended pregnancy								
No	-		-		-		-	
Yes	1.18	(0.54–2.54)	1.85	(0.82–4.21)	0.94	(0.63–1.42)	0.40 **	(0.21–0.75)
FTM’s highest level of schooling								
None/primary/secondary incomplete	-		-		-		-	
Secondary complete or higher	1.12	(0.61–2.07)	1.03	(0.65–1.65)	1.06	(0.71–1.59)	1.04	(0.66–1.65)
Household wealth								
Low	-		-		-		-	
Medium	0.84	(0.47–1.49)	1.18	(0.77–1.82)	0.90	(0.56–1.47)	0.72	(0.41–1.26)
High	1.15	(0.60–2.19)	0.73	(0.46–1.16)	1.33	(0.81–2.18)	0.99	(0.56–1.76)
Worked last year								
No	-		-		-		-	
Yes	0.97	(0.55–1.71)	1.60 *	(1.06–2.40)	1.15	(0.79–1.68)	1.09	(0.70–1.69)
Watches TV at least once a week								
No	-		-		-		-	
Yes	0.71	(0.41–1.23)	0.79	(0.53–1.17)	0.96	(0.63–1.46)	1.40	(0.86–2.29)
Intervention health zone								
Bembu	-		-		-		-	
Kingasani	1.10	(0.48–2.50)	0.46 *	(0.24–0.87)	0.93	(0.47–1.83)	0.28 **	(0.12–0.66)
Lemba	0.20 **	(0.08–0.54)	0.28 ***	(0.13–0.57)	0.83	(0.38–1.81)	0.15 ***	(0.06–0.40)
Masina1	0.28 **	(0.11–0.70)	0.62	(0.31–1.21)	1.08	(0.55–2.12)	0.35 *	(0.15–0.81)
Matete	0.52	(0.18–1.48)	0.30 **	(0.14–0.63)	1.38	(0.65–2.91)	0.39 *	(0.16–0.96)
Ndjili	0.41	(0.16–1.02)	0.67	(0.35–1.28)	1.10	(0.53–2.26)	0.34 *	(0.15–0.80)
N	315		540		497		398	

* *p* < 0.05, ** *p* < 0.01, *** *p* < 0.001. Source: Momentum 2018 Baseline Survey and 2020 Endline Survey.

**Table A3:** Multivariable logistic regression model of timely postpartum or postnatal care visit among first-time mothers aged 15–24 years, Kinshasa, DRC 2020 by age and relationship status.

Variables	Model 1							
	PPC/PNC							
	15–19 Years				20–24 Years			
	In-Union		Not In-Union		In-Union		Not On-Union	
	Adjusted Odds Ratio	95% CI	Adjusted Odds Ratio	95% CI	Adjusted Odds Ratio	95% CI	Adjusted Odds Ratio	95% CI
Decision-making autonomy								
Low	-		-		-		-	
Medium	1.12	(0.64–1.97)	0.68	(0.44–1.04)	0.86	(0.56–1.32)	0.81	(0.48–1.37)
High	1.18	(0.62–2.25)	0.94	(0.60–1.48)	0.96	(0.58–1.60)	0.58	(0.34–1.01)
Autonomy: Postpartum contraception								
No	-		-		-		-	
Yes	0.71	(0.43–1.17)	1.07	(0.74–1.55)	1.19	(0.80–1.76)	0.78	(0.50–1.22)
Autonomy: Kangaroo Mother Care								
No	-		-		-		-	
Yes	1.59	(0.91–2.78)	1.84 **	(1.19–2.85)	1.96 **	(1.21–3.17)	1.33	(0.79–2.23))
Autonomy: Breastfeeding								
No	-		-		-		-	
Yes	1.20	(0.70–2.07)	1.00	(0.67–1.50)	1.18	(0.76–1.85)	1.23	(0.78–1.95)
Male involvement in maternal care								
Low	-		-		-		-	
Medium	0.66	(0.36–1.24)	1.21	(0.78–1.86)	0.72	(0.43–1.22)	0.84	(0.50–1.39)
High	0.50 *	(0.26–0.98)	0.88	(0.53–1.46)	0.48*	(0.27–0.85)	0.53 *	(0.29–0.99)
Male involvement in childcare								
Low	-		-		-		-	
Medium	1.91	(0.99–3.68)	1.20	(0.80–1.81)	1.80 *	(1.07–3.04)	1.12	(0.68–1.84)
High	1.61	(0.77–3.37)	2.39 **	(1.31–4.37)	2.29 **	(1.29–4.05)	2.06 *	(1.08–3.90)
Unintended pregnancy								
No	-		-		-		-	
Yes	1.11	(0.52–2.39)	1.60	(0.70–3.69)	0.87	(0.58–1.30)	0.49 *	(0.27–0.90)
FTM’s highest level of schooling								
None/primary/secondary incomplete	-		-		-		-	
Secondary complete or higher	1.50	(0.82–2.77)	0.97	(0.61–1.54)	1.25	(0.83–1.88)	0.97	(0.71–1.74)
Household wealth								
Low	-		-		-		-	
Medium	0.99	(0.55–1.82)	0.94	(0.60–1.46)	0.82	(0.50–1.34)	0.60	(0.33–1.07)
High	1.17	(0.60–2.30)	0.72	(0.45–1.15)	1.10	(0.66–1.84)	0.86	(0.47–1.58)
Worked last year								
No	-		-		-		-	
Yes	1.09	(0.63–1.90)	1.49	(0.99–2.24)	1.17	(0.80–1.70)	1.11	(0.71–1.74)
Watches TV at least once a week								
No	-		-		-		-	
Yes	0.63	(0.37–1.10)	0.87	(0.59–1.29)	0.99	(0.65–1.49)	1.56	(0.97–2.56)
Intervention health zone								
Bembu	-		-		-		-	
Kingasani	1.02	(0.44–2.35)	0.58	(0.31–1.11)	1.00	(0.51–1.97)	0.33 *	(0.14–0.78)
Lemba	0.26 **	(0.10–0.68)	0.39 **	(0.19–0.79)	1.00	(0.46–2.13)	0.26 **	(0.10–0.68)
Masina1	0.31 *	(0.13–0.77)	0.61	(0.31–1.20)	1.19	(0.61–2.34)	0.41 *	(0.17–0.98)
Matete	0.71	(0.25–2.01)	0.53	(0.26–1.07)	1.89	(0.89–4.02)	0.54	(0.21–1.36)
Ndjili	0.43	(0.17–1.06)	0.80	(0.42–1.55)	1.63	(0.79–3.36)	0.39 *	(0.17–0.93)
N	315		540		497		398	

* *p* < 0.05, ** *p* < 0.01. Source: Momentum 2018 Baseline Survey and 2020 Endline Survey.

**Table A4:** Multivariable logistic regression model of timely postpartum care visit among first-time mothers aged 15–24 years, Kinshasa, DRC 2020 with 42-day cut-off.

Variables	Model 1					
	Postpartum Care		Postnatal Care		PPC/PNC	
	Adjusted Odds Ratio	95% CI	Adjusted Odds Ratio	95% CI	Adjusted Odds Ratio	95% CI
Decision-making autonomy						
Low	Ref		Ref		Ref	
Medium	1.24	(0.95–1.63)	1.18	(0.92–1.51)	1.14	(0.90–1.43)
High	1.19	(0.88–1.61)	1.18	(0.90–1.55)	1.14	(0.88–1.47)
Autonomy: Postpartum contraception						
No	Ref		Ref		Ref	
Yes	1.10	(0.86–1.39)	1.13	(0.84–1.30)	1.03	(0.84–1.26)
Autonomy: Kangaroo Mother Care						
No	Ref		Ref		Ref	
Yes	1.35 *	(1.01–1.81)	1.48 **	(1.13–1.93)	1.61 ***	(1.26–2.07)
Autonomy: Exclusive Breastfeeding						
No	Ref		Ref		Ref	
Yes	0.90	(0.70–1.17)	1.01	(0.80–1.29)	1.17	(0.93–1.45)
Male involvement in maternal care						
Low	Ref		Ref		Ref	
Medium	0.80	(0.60–1.08)	0.86	(0.66–1.12)	1.00	(0.78–1.28)
High	0.91	(0.66–1.26)	0.60 **	(0.44–0.82)	0.80	(0.60–1.05)
Male involvement in childcare						
Low	Ref		Ref		Ref	
Medium	1.07	(0.80–1.42)	1.08	(0.83–1.41)	1.04	(0.81–1.32)
High	1.34	(0.96–1.88)	1.29	(0.94–1.76)	1.17	(0.87–1.57)
Unintended pregnancy						
No	Ref		Ref		Ref	
Yes	0.86	(0.62–1.20)	0.99	(0.73–1.34)	0.87	(0.66–1.16)
Age group						
15–19	Ref		Ref		Ref	
20–24	1.05	(0.81–1.38)	1.02	(0.80–1.31)	0.99	(0.78–1.24)
In-union						
No	Ref		Ref		Ref	
Yes	0.84	(0.65–1.08)	0.97	(0.77–1.22)	0.82	(0.66–1.01)
FTM’s highest level of schooling						
None/primary/secondary incomplete	Ref		Ref		Ref	
Secondary complete or higher	1.20	(0.92–1.57)	1.18	(0.92–1.51)	1.22	(0.97–1.54)
Household wealth						
Low	Ref		Ref		Ref	
Medium	0.82	(0.60–1.11)	1.16	(0.88–1.52)	0.99	(0.76–1.27)
High	1.09	(0.80–1.48)	1.12	(0.84–1.50)	1.09	(0.84–1.42)
Worked last year						
No	Ref		Ref		Ref	
Yes	1.18	(0.92–1.50)	1.31 *	(1.05–1.64)	1.25 *	(1.01–1.54)
Watches TV at least once a week						
No	Ref		Ref		Ref	
Yes	1.11	(0.85–1.45)	0.92	(0.72–1.17)	1.01	(0.81–1.26)
Intervention health zone						
Comparison	Ref		Ref		Ref	
Intervention	1.54 ***	(1.21–1.95)	1.06	(0.86–1.32)	1.16	(0.95–1.42)
N	1750		1750		1750	

* *p* < 0.05, ** *p* < 0.01, *** *p* < 0.001. Source: Momentum 2018 Female Baseline Survey and 2020 Female Endline Survey.

## Appendix C

Decision-making autonomy index questions

1. Would you say that when to start seeking antenatal care for the pregnancy is (was) mainly your decision, mainly your husband’s/partner’s decision, someone else’s decision, or do (did) you and your husband/partner decide together?
2. Would you say that the number of antenatal visits you make (made) is (was), mainly your husband’s/partner’s decision, someone else’s decision, or do (did) you and your husband/partner decide together?
3. Would you say that where to deliver the baby is (was) mainly your decision, mainly your husband’s/partner’s decision, someone else’s decision, or do (did) you and your husband/partner decide together?
4. Would you say that how long to wait after childbirth before attempting another pregnancy is (was) mainly your decision, mainly your husband’s/partner’s decision, someone else’s decision, or do (did) you and your husband/partner decide together?
5. Would you say that how soon to start breastfeeding your newborn after childbirth is (was) mainly your decision, mainly your husband’s/partner’s decision, someone else’s decision, or do (did) you and your husband/partner decide together?
6. Would you say that whether to practice exclusive breastfeeding is (was) mainly your decision, mainly your husband’s/partner’s decision, someone else’s decision, or do (did) you and your husband/partner decide together?
7. Would you say that how to take care of the baby’s umbilical cord is (was) mainly your decision, mainly your husband’s/partner’s decision, someone else’s decision, or do (did) you and your husband/partner decide together?
8. Would you say that when to seek care and treatment for danger signs of the mother or newborn is (was) mainly your decision, mainly your husband’s/partner’s decision, someone else’s decision, or do (did) you and your husband/partner decide together?

Please tell me if your husband/partner was present at the following occasions during pregnancy and labor?

1. Pregnancy test or when the pregnancy was confirmed?
2. One or more antenatal visits?
3. One or more group education classes about pregnancy and fatherhood?
4. At childbirth/delivery or when you lost your pregnancy?

Please tell me if your husband/partner was involved in the following things:

1. Finding information about the pregnancy?
2. Making decisions about antenatal care?
3. Making a birth plan?
4. Saving money for emergencies?
5. Arranging transport for delivery?
6. Deciding on skilled attendance at delivery?
7. Arranging for blood donor?
8. Encourage exclusive breastfeeding?

Please tell me if the father of the baby was involved a great deal, a bit, or not at all in the following activities after the baby was born?

1. Changing the baby’s diapers?
2. Helping/supporting feeding?
3. Helping with baby cries?
4. Bathing the baby?
5. Playing with the baby?
6. Looking after the baby when the mother goes out or is at work?
7. Cooking food?
8. Washing the baby’s clothes?
